# Lipidic Formulations Inspired by COVID Vaccines as Smart Coatings to Enhance Nanoparticle-Based Cancer Therapy

**DOI:** 10.3390/nano13152250

**Published:** 2023-08-03

**Authors:** Marzia Conte, Marco Carofiglio, Giada Rosso, Valentina Cauda

**Affiliations:** Department of Applied Science and Technology, Politecnico di Torino, Corso Duca degli Abruzzi 24, 10129 Torino, Italy; marzia.conte@polito.it (M.C.); marco.carofiglio@polito.it (M.C.); giada.rosso@polito.it (G.R.)

**Keywords:** lipid nanoparticles, phospholipid shell, zinc oxide nanoparticles, biomimetic nanoparticles, solvent exchange method

## Abstract

Recent advances in nanomedicine have led to the introduction and subsequent establishment of nanoparticles in cancer treatment and diagnosis. Nonetheless, their application is still hindered by a series of challenges related to their biocompatibility and biodistribution. In this paper, we take inspiration from the recently produced and widely spread COVID vaccines, based on the combinational use of ionizable solid lipid nanoparticles, cholesterol, PEGylated lipids, and neutral lipids able to incorporate mRNA fragments. Here, we focus on the implementation of a lipidic formulation meant to be used as a smart coating of solid-state nanoparticles. The composition of this formulation is finely tuned to ensure efficient and stable shielding of the cargo. The resulting shell is a highly customized tool that enables the possibility of further functionalizations with targeting agents, peptides, antibodies, and fluorescent moieties for future in vitro and in vivo tests and validations. Finally, as a proof of concept, zinc oxide nanoparticles doped with iron and successively coated with this lipidic formulation are tested in a pancreatic cancer cell line, BxPC-3. The results show an astonishing increase in cell viability with respect to the same uncoated nanoparticles. The preliminary results presented here pave the way towards many different therapeutic approaches based on the massive presence of highly biostable and well-tolerated nanoparticles in tumor tissues, such as sonodynamic therapy, photodynamic therapy, hyperthermia, and diagnosis by means of magnetic resonance imaging.

## 1. Introduction

Liposomes have been an object of research since the 1960s, when they were first introduced as innovative and efficient drug delivery systems (DDSs), able to overcome the low therapeutic indexes of traditional chemotherapy [1,2]. Since that time, the use of liposomes and, more generally, lipid molecules has interested different branches of nanomedicine. The primary aim is to address the numerous challenges raised by the need for efficient delivery of both therapeutics and genetic material to specific target sites. These applications range from cancer therapy to gene therapy and vaccination [3].

In this latter case, the notorious COVID-19 pandemic certainly boosted worldwide the search for a robust solution to provide rapid and unprecedented mass production of vaccines. When it came to deciding the way to convey immunization to hundreds of millions of people, different alternatives were taken into account, and finally, mRNA vaccines were selected [4]. In order to guarantee prolonged blood circulation and precious cargo protection, the scientific community agreed on the use of already well-known ionizable lipid-based nanoparticles (LNPs) [5]. These LNPs were implemented by different pharmaceutical companies in parallel to guarantee a shortening of the conventionally long validation process by competent regulatory agencies [6]. In particular, two main vaccines debuted on the market almost simultaneously after receiving the first historical authorization by the FDA (Food and Drug Administration) and EMA (European Medicines Agency) for emergency use [7]: those produced by BioNTech/Pfizer (BNT162b2) and those realized by Moderna (mRNA-1273). Leaving aside the differences between the two in terms of antigen choice and mRNA design and modification, we focused on the implementation of the delivery system employed in these two case studies. 

Briefly, both were formulated through microfluidic mixing in a scalable production system. The formulations exploit the ability of their proprietary and innovative ionizable lipids, included in the formulation, to acquire a positive charge under some specific pH conditions. This enables an electrostatic complexation of the negatively charged mRNA. A further part of the process carried out at neutral pH allowed the formation of uncharged solid-core lipid nanoparticles densely packaging mRNA and able to release their cargo via endosomal escape once internalized by cells, according to mechanisms still not fully understood [8]. The compositions of these LNPs were initially hypothesized according to previous works of the developers, which had employed the same type of lipid nanoparticles in Phase I/II studies [9,10,11]. Subsequently, the compositions were disclosed or confirmed in official patents published by Moderna [12,13] and Pfizer [14], respectively. In detail, they comprised a combination of a new class of ionizable cationic lipids, a neutral phospholipid, cholesterol, and a polyethylene glycol (PEG) lipid [15]. The selection of their molar ratios, composition, and choice was the result of extensive trials conducted over several years in the context of delivery systems for mRNA vaccines [16]. Various strategies, such as the use of cationic nanoemulsions [17], nanostructured lipid nanoparticles [18], cationic polymers [19], and LNPs encoding antibodies [20], were reported as strategies to efficiently deliver mRNA strands in vaccines. 

The underlying common principle of all these studies was the interaction between the negatively charged nucleic acid and the positively charged lipids composing the carrier in question to guarantee dense packaging and enhanced protection of the cargo. This notion was our starting point to develop COVID vaccine-inspired permanently charged lipidic formulations aimed at being used as smart coatings of nanoparticles or even drug carriers of charged moieties for cancer applications [21]. At first, we decided to put aside the use of ionizable lipids in favor of a less complex system, replacing them with permanently charged positive or negative lipids, to obtain a first idea of their behavior when electrostatically interacting with the cargoes. Acuitas patent and the above-mentioned first speculations on the composition of the LNPs were thoroughly studied to establish the molar ratios of the various components of the shell, namely charged lipids, neutral lipids, cholesterol, and PEGylated lipids, which were set at 50:10:38:5:1:5. 

Different techniques and solvents were employed to realize the liposomes and characterize them. Afterward, a precise protocol was established to achieve a reproducible and successful coating of metal oxide nanoparticles (NPs). Firstly, a mini extruder was used to obtain monodisperse and highly stable uncharged and charged liposomes, employable as drug carriers for charged moieties and applicable to cancer treatments as well as gene therapy [22]. Then, in order to successfully use the lipidic formulations as coatings for charged solid inorganic nanoparticles, another methodology was implemented and optimized, namely the solvent exchange technique. As diffusely discussed in previous reviews on the topic [2], the decoration and coating of metal oxide and, more generally, solid-state inorganic nanoparticles with artificially or naturally derived phospholipidic bilayers is the main path taken to confer them a higher biostability and biocompatibility. This process aims to guarantee a better interaction of the nanomaterials in the object with the organism by drastically reducing their toxicity and immunogenic response [23,24]. 

With respect to the existing body of research already published on this specific topic (namely lipid-coated zinc oxide nanoparticles for cancer applications) by either our group [25,26] or other research teams [27], the importance and originality of this study consist in the use of an innovative and highly customizable lipidic shell. This lipidic shell’s interaction with the cargo is inspired by the most recent and widespread—and therefore broadly tried and tested—lipid nanoparticle formulations. 

As a proof of concept, these lipid-coated NPs were tested against a pancreatic cancer cell line (BxPC-3) to show their increased biostability and biocompatibility compared to uncoated nanoparticles. The nanoparticles at the core of the nanoconstructs, designed and already applied in previous works [28,29], are zinc oxide NPs doped with iron, whose effect has been extensively shown to increase the tolerability and decrease the dissolution of the NPs in biological systems [30,31]. 

**Figure 1:**
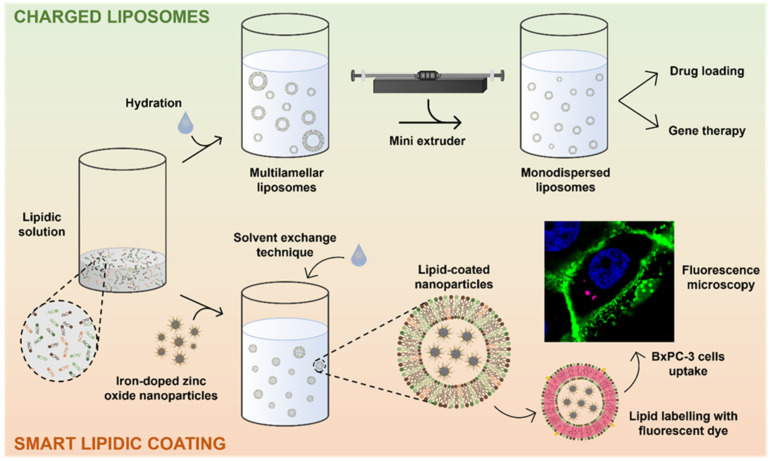
Scheme reporting the formation of the extruded liposomes and the solvent exchange technique employed to coat metal oxide nanoparticles.

To fully exploit the potential of the lipidic shell, designed to be further functionalized with targeting peptides or fragmented antibodies for theranostic or immunotherapeutic purposes, the pancreatic cancer targeting peptide, CKAAKN, was conjugated to the lipidic shell. The results showed an increased cell uptake with respect to lipid-coated NPs when administered to BxPC-3 cells. A scheme of the procedures described in this work is reported in Figure 1.

## 2. Materials and Methods

### 2.1. Lipidic Formulations

All the employed lipids were purchased by Avanti Polar Lipids Inc. (Birmingham, AL, USA): DOPA (18:1 PA, 1,2-dioleoyl-sn-glycero-3-phosphate (sodium salt), chloroform solution); DOPC (18:1 (Δ9-Cis) PC (DOPC), 1,2-dioleoyl-sn-glycero-3-phosphocholine, chloroform solution); DSPE-PEG(2000) Amine (1,2-distearoyl-sn-glycero-3-phosphoethanolamine-N-[amino(polyethylene glycol)-2000] (ammonium salt)); and DSPE-PEG(2000) Maleimide (1,2-distearoyl-sn-glycero-3-phosphoethanolamine-N-[Maleimide(polyethylene glycol)-2000] (ammonium salt)); the cholesterol solution in chloroform was purchased by Sigma-Aldrich (St. Louis, MO, USA). Three different lipidic formulations were realized: a neutral one, hereinafter called Formulation 2C (Form2C for brevity), a positive one, called Formulation 3C^+^ (Form3C^+^), and a negative one, Formulation 3C^−^ (Form3C^−^). Table 1 reports the types of lipids and the molar ratios employed to realize them.

According to these proportions, the lipidic solutions (either in chloroform as provided by the manufacturers or in methanol for the PEGylated lipids, supplied in powder and later dispersed in this solvent) were put in a glass vial to let the solvent evaporate overnight under vacuum. From this common starting point, different strands of research have been developed in order to develop various methodologies and identify the best protocol for future experiments. The main differences consisted of the solvent employed for the redispersion of lipids and the technique carried out to obtain the liposomes. 

### 2.2. Mini Extruder Technique

The first experiment involved the use of a mini-extruder purchased by Avanti Polar Lipids. The dried lipids were rehydrated in a solution made up of bidistilled water (obtained from a Direct Q3 system, Millipore, Burlington, MA, USA) and physiological solution (Galenica Senese S.r.l., Monteroni D’arbia, Italy) to allow the formation of liposomes. The obtained multilamellar liposomes were characterized in terms of hydrodynamic size (Dynamic Light Scattering, DLS) and zeta potential using a Zetasizer Nano ZS90 (Malvern Panalytical, Malvern, UK) by diluting a volume corresponding to 100 µg of liposomes in 1 mL of bidistilled water. Afterward, they were subjected to a total of 11 passages through the membrane of the mini-extruder (whose pores had a diameter of 100 nm) at a temperature of 60 °C. The aim of the extrusion was to obtain well-dispersed liposomes, reduce their size range, and improve their stability over time. After the extrusion process, the previously mentioned characterizations were carried out again. Moreover, a Nanoparticle Tracking Analysis (NTA) using the NanoSight NS300 from Malvern Panalytical was performed on the extruded samples. Once the success of the extrusion technique was assessed, the obtained liposomes were stored at +4 °C and analyzed in terms of hydrodynamic size and zeta potential in water after 10 days to prove their stability over time and their ability to maintain their charge unaltered, comparing their behavior before and after the extrusion. The established protocol was easily adaptable for the future incorporation of drugs. The drugs could be dissolved in either organic solvents (therefore dried with the lipids) or in the aqueous phase and added during the rehydration step, right before the extrusion. 

However, such an approach was not suitable for the incorporation of nanoparticles into the liposomes due to the small dimension of the pores of the extruder and the fact that the NPs would remain stuck in the filter. For this reason, another route was chosen to incorporate them into the lipidic shell, namely an adaptation of the previously reported solvent exchange technique [24,32]. The choice of the best formulation to be used as a coating for our positively charged iron-doped zinc oxide NPs (whose fabrication and physio–chemical characterizations have been thoroughly described in previous work [28] by Carofiglio et al.) naturally fell on Form3C^−^ to exploit the electrostatic interaction between cargo and shell. Moreover, we kept calling this formulation Form3C^−^ throughout the manuscript since its name refers to both the number of lipidic components employed (i.e., three different phospholipids) and the presence of cholesterol (C) in the shell.

### 2.3. Solvent Exchange Method

**Figure 2:**
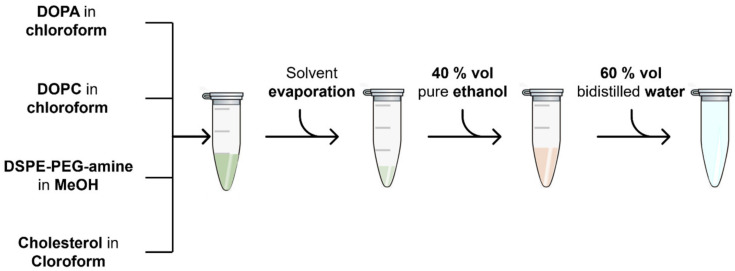
Process scheme of the preparation of the lipidic formulations.

Briefly, after the lipids were left dry overnight under vacuum, they were hydrated with a solution consisting of ethanol (99%, Sigma Aldrich, Burlington, MA, USA) and bidistilled water in a volume proportion of 40–60% (Figure 2). This ratio was carefully studied so as to avoid any unwanted self-assembly of the lipids. For this purpose, ethanol was added as the first solvent. The resulting lipid dispersion, whose color would turn whitish after the addition of water, was stable and kept at 4 °C for the following steps.

**Figure 3:**
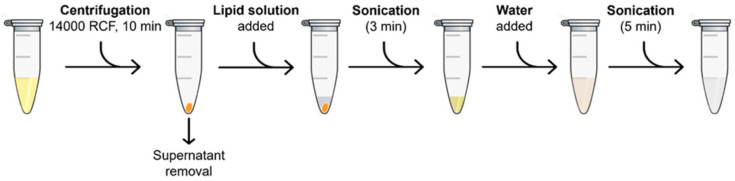
Process scheme of the NPs coating with the lipidic formulations.

The coating process consisted of pelleting a certain amount (defined later for the optimized NPs coating protocol) of nanoparticles from the ethanolic stock by centrifuging them at 14,000× *g* for 10 min and removing the supernatant. Then, a volume of lipid solution was added to the pellet, and a first step of sonication (3 min) employing an ultrasound bath (59 kHz, Branson 3800 CPXH, Branson Ultrasonics Corporation, Brookfield, CT, USA) was performed to allow a good dispersion of the nanoparticles and the lipids. Afterward, bidistilled water was suddenly added to the mixture, and a second cycle of sonication (5 min) was performed. From the resulting solution, the volume corresponding to 100 μg of NPs was withdrawn, added to 900 μL of bidistilled water, and analyzed via DLS and zeta potential. Figure 3 reports the process scheme just described.

After several trials, described in Section 3 and in the Supporting Information (S.I.) sections, an optimal ratio between the weight of the NPs and the weight of the lipids employed for the lipidic coating was established and used for all the following experiments.

### 2.4. Cytotoxicity Study on BxPC-3 Cells

As a proof of concept, the lipid-coated nanoparticles were tested against a human pancreatic cancer cell line, BxPC-3 (ATCC CRL-1687). The cell line was cultivated with RPMI 1640 medium (ATCC) supplemented with 10% heat-inactivated FBS (ATCC), 100 μg/mL streptomycin, and 100 units/mL penicillin (Sigma Aldrich) and grown at 37 °C with a 5% CO_2_ atmosphere. Briefly, 2500 cells were seeded in each well of a 96-well culture plate (TC-Treated, Corning, Corning, NY, USA) and incubated for 24 h at 37 °C in a 5% CO_2_ atmosphere. Then, the cell culture medium was replaced with fresh medium containing different doses of either naked (10, 15, 20, and 30 μg/mL) or lipid-coated (10, 15, 20, 30, 40, 50, 75, 100, and 150 μg/mL) NPs. To prepare naked NP dispersions, the NPs were first sonicated in their ethanolic stock solution for 10 min. Then, the volume corresponding to the correct amount for each sample was withdrawn from the stock solution and put in a cell culture medium. For coated NP dispersions, the NPs were simply withdrawn from a freshly prepared aqueous stock solution prepared as in Section 2.3 and directly dispersed in the correct amount in RPMI. Background wells containing just NPs dispersed in RPMI were also prepared. After 24, 48, and 72 h of incubation with the NPs, the WST-1 proliferation assay (Roche, Basel, Switzerland) was performed to assess cell viability. Briefly, 10 μL of WST-1 reagent was added to the wells 2 h prior to each time step and incubated at 37 °C in a 5% CO_2_ atmosphere. Then, the absorbance was measured with a Multiskan GO microplate spectrophotometer (Thermo Fisher Scientific, Waltham, MA, USA) at 450 nm, using 620 nm as a reference wavelength. All the background values were subtracted from the absorbance value of each sample, and the resulting measurements were compared to the control cells. The control cells were incubated in simple RPMI and were used as a reference for 100% viability. All the measurements carried out throughout the work were taken at least in triplicate, and the statistical one-way or two-way ANOVA analysis of variance was performed with Origin V9.0 software. 

### 2.5. Functionalizations and Targeting Peptide

One of the key features of the lipidic formulation here implemented is its customizable value, thanks to the presence of the DSPE-PEG (2000) Maleimide lipid in the shell. In fact, using simple chemical reactions, it is possible to attach species such as peptides and fragments of antibodies, exploiting the cysteine residue of the Maleimide compound. To assess the ability of the nanoconstructs to target pancreatic cancer cells, a custom-made targeting peptide, namely CKAAKN, was purchased by Bio-Fab Research and linked to the DSPE-PEG (2000) Maleimide lipid, as reported hereinafter.

#### 2.5.1. DSPE-PEG (2000) Maleimide Coupling to Peptide

Briefly, the DSPE-PEG (2000) Maleimide lipid and the CKAAKN peptide (molar ratio 3:1) were dissolved in N, N-Dimethylformamide (DMF, Sigma Aldrich) at a concentration of 37.5 mM and 50 mM, respectively. Then, the peptide solution was diluted in 0.1 M sodium phosphate buffer (PBS, pH 7.4), and the DSPE-PEG (2000) Maleimide solution was added. This resulted in a final reaction mixture of 1:1 DMF/PBS, a 5 mM peptide, and a 15 mM lipid concentration. The reaction was allowed to proceed at room temperature for 1 h. The resulting mixture (DSPE-PEG (2000)-CKAAKN, hereinafter called functional lipid for brevity) was stored at −20 °C and kept as stock. Before using it in the lipidic formulation, it was diluted in ethanol (1:10 dilution) in a molar ratio of 0.1% (Figure S1, Supplementary Information). To assess the correct coating of the nanoparticles, a custom-made CKAAKN peptide already linked to fluorescein isothiocyanate (FITC) was purchased by Bio-Fab Research. This peptide was then linked to the DSPE-PEG (2000) Maleimide lipid for imaging purposes, following the same procedure reported above (this other compound, DSPE-PEG (2000)-CKAAKN-FITC, will be referred to as a fluorescent functional lipid).

#### 2.5.2. Colocalization with Fluorescence Microscopy

In order to perform the qualitative colocalization studies by means of fluorescence microscopy, the process of lipid coating was carried out on previously labeled NPs. Briefly, NPs were withdrawn from the ethanolic stock, and Atto647 NHS ester (ThermoFisher, Waltham, MA, USA) was added (2 µg/mg of NPs) in the dark and stirred overnight. Then, the NPs were washed three times via centrifugation at 14,000× *g* for 10 min and redistribution in ethanol. Then, the protocol for lipid drying and coating was followed, taking care to add the previously prepared fluorescent functional lipid to the formulation in a molar ratio of 0.1% (Figure S1, Supplementary Information). For the fluorescence microscopy assay, the obtained nanoconstructs were further diluted in water; 2 μL of the sample was spotted on a microscope slide, coated with a cover slide, and let dry in the dark. The samples were imaged with a wide-field inverted fluorescence microscope (Eclipse TiE from Nikon) equipped with a 100× objective (NA = 1.30). Then, employing the colocalization tool of the NIS NIS-Elements AR-SP software 4.50 (Nikon, Tokyo, Japan) and adapting some optimized settings reported in previous work [33], the percentage of colocalization was evaluated.

### 2.6. Internalization Studies

To show the internalization of the NPs inside BxPC-3 cells, two distinct experiments were carried out once the optimal safe concentration of NPs was established (as described in Section 3, 50 μg/mL). First, an uptake study employed a flow cytometer, followed by a qualitative fluorescence microscopy analysis.

### 2.7. Flow Cytometry

For the flow cytometry study, BxPC-3 cells (3 × 10^5^ cells in 500 μL of cell culture media) were seeded in 24-well culture plates (TC treated, Thermo Fisher) and incubated for 24 h at 37 °C with a 5% CO_2_ atmosphere. NPs were previously labeled with Atto647 NHS ester as described above and then coated with three different coatings, namely Form3C^−^, Form3C^−^ containing the CKAAKN peptide, and finally Form3C^−^ with the CKAAKN-FITC peptide, to compare the effect of the targeting peptide in terms of internalization. After the standard coating process, the nanoconstructs were incubated with DiD’ dye (DiIC18(5) solid (1,1′-Dioctadecyl-3,3,3′,3′-Tetramethylindodicarbocyanine, 4-Chlorobenzenesulfonate Salt), Invitrogen) previously dissolved in dimethyl sulfoxide (DMSO, Sigma Aldrich) at 37 °C for 30 min under continuous shaking and then dispersed in cell culture medium and administered to cells. After a further 24 h of incubation, cells were washed twice with PBS to remove the non-internalized NPs and then detached by trypsinization, collected, and centrifuged at 130× *g* for 5 min. Finally, they were dispersed in PBS and analyzed with a Guava Easycyte 6-2L flow cytometer (Merck Millipore, Burlington, MA, USA). The number of events corresponding to the analyzed cells was acquired and analyzed as described in previous works [34]. The analyses were performed with Incyte Software (Merck Millipore), while graphs were obtained through FCS Express 6 Software (DeNovo Software) and Origin V9.0 software (OriginLab, Northampton, MA, USA). Tests were performed in triplicate, and ANOVA analysis of variance was performed with Origin V9.0 software.

### 2.8. Fluorescence Microscopy

To provide qualitative proof of NP internalization into cells and to locate their position, fluorescence microscopy analyses were performed on BxPC-3 cells 24 h after NP administration employing spinning-disk confocal fluorescence microscopy (Ti2 Nikon equipped with a crest large FOV laser and a 60× PlanAPO objective, NA = 1.40). Briefly, 1 × 10^4^ cells were seeded into 8-well chamber slides (Nunc Lab-Tek II CC2 Chamber Slide System, Thermo Fisher Scientific) with 250 μL of complete cell culture medium. After 24 h of incubation, Form3C^−^-CKAAKN NPs, previously labeled with DiD as described above, were administered to cells at a concentration of 50 μg/mL. After a further 24 h, cells were fixed by replacing the cell culture medium with 150 μL of Image-IT fixative solution (Thermo Fisher). After 10 min at room temperature, they were washed twice with PBS, and the membranes were stained with 250 μL of PBS containing wheat germ agglutinin conjugated with an Alexa Fluor 488 dye (WGA-488, Thermo Fisher) at a concentration of 2.5 μg/mL for 10 min at 37 °C in a 5% CO_2_ atmosphere. Again, cells were washed twice with PBS, and Hoechst (Thermo Fisher), at a concentration of 0.3 μg/mL in PBS, was administered for nuclei staining. After a further 5 min in normal cell culture conditions, they were washed twice with PBS, and live cell imaging (LCI, Molecular Probes, Eugene, OR, USA) solution was added. The obtained samples were analyzed right after the staining.

### 2.9. Hemocompatibility Test

To assess the effect of the lipidic shell on improving the NPs’ hemocompatibility, a plasma recalcification test was conducted following an established protocol [35,36,37]. The lipidic coating process was carried out as described in Section 2.3, but the whole procedure took place under a sterile hood. Naked and the so-obtained Form3C^−^ NPs were both resuspended in 0.1 µm filtered physiological solution (0.9% NaCl *w*/*w* water solution) at a final concentration of 50 and 100 µg/mL, while pure physiological solution was used as a control sample. A total of 6 wells per sample of a 96-well plate were filled with 75 µL of human citrated plasma (Human Recovered Plasma Pooled-frozen—NaCitrate from ZenBio, Durham, NC, USA) pre-heated at 37 °C. Then, 75 µL of the sample (either control or zinc oxide NPs, naked or coated, at the two different concentrations) was added to each well. After 5 min of incubation at 37 °C, 150 µL of 25 mM calcium chloride (CaCl_2_) was quickly added in 3 wells for each sample to induce the coagulation of plasma. Right afterward, the absorbance at 405 nm was periodically measured by inserting the plate in a 37 °C pre-heated UV–Vis spectrophotometer. In detail, every 30 s, measurements were carried out for a total of 45 min at an incubation temperature of 37 °C. Three independent experiments were conducted. The resulting coagulation times, determined as reported in detail in previous work [38], were averaged.

## 3. Results and Discussion

This section may be divided into subheadings. It should provide a concise and precise description of the experimental results, their interpretation, and the experimental conclusions that can be drawn.

### 3.1. Mini Extruder Technique

**Figure 4:**
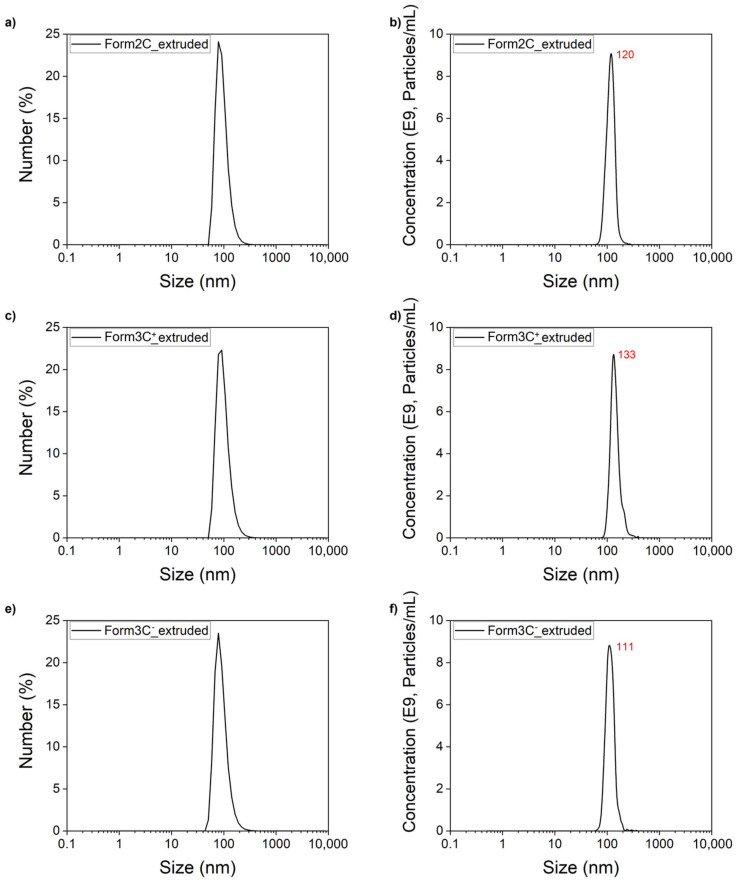
DLS size (**a**,**c**,**e**) and NTA (**b**,**d**,**f**) measurements of the three liposomal formulations after the extrusion process.

The results displayed in Table 2 and Figure 4 show all three lipidic formulations before and after the extrusion. As reported, the extrusion process positively affected the PDI and the size of the liposomes, which were heavily reduced in all cases. In fact, the passages through the membrane of the mini-extruder had the effect of creating unilamellar vesicles via the application of shear stress combined with the heating of the whole system over the phase transition temperature of the involved lipids [39]. Moreover, in accordance with what was expected, the values of the zeta potential reflected the employment of neutral, positive, and negative lipids in each formulation. The effect of the extrusion on the charge of the liposomes was not as marked as for the size and the PDI, but the standard deviation of the measurements was reduced for all the samples. To confirm the results obtained via the DLS, a NTA measurement was carried out for all the extruded samples. The NTA measurements showed monodisperse liposomes with sizes comparable to those obtained with the DLS measurements.

Overall, these results confirmed that the mini extruder technique could successfully be used to realize monodispersed liposomes regardless of the charge of the lipids, with robust and predictable results suitable for further functionalizations or cargo loadings.

#### 3.1.1. Stability Assay

**Figure 5:**
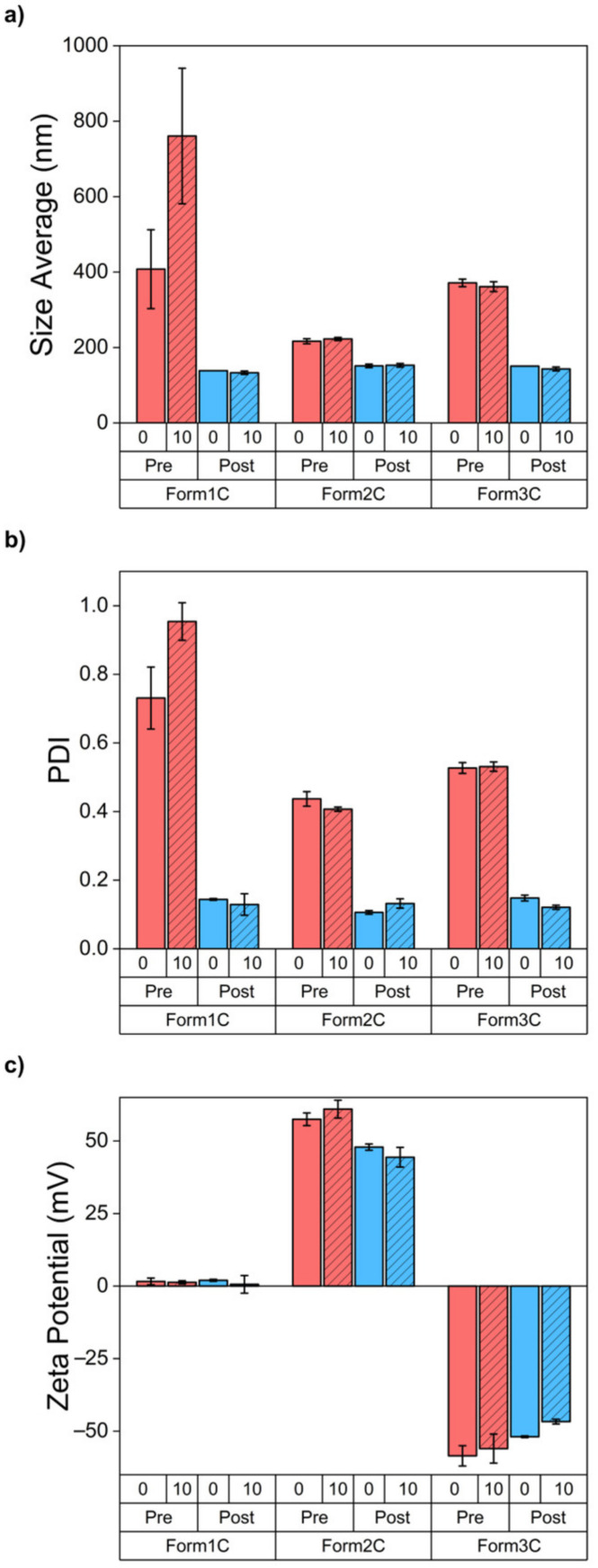
Comparison between size average (**a**), PDI (**b**), and zeta potential (**c**) measurements of the three liposomal formulations pre- and post-extrusion and stability assay after 10 days of storage at +4 °C.

After the previously reported characterizations, the samples were stored at +4 °C and analyzed after 10 days to assess their stability via DLS and zeta potential measurements. As shown by Figure 5 and Table S1, all the extruded samples were extremely stable even after 10 days of storage since PDI, size, and zeta potential remained almost unchanged over time. 

These stability results were of utmost importance since they proved that the lipidic formulations could be potentially prepared in stocks and kept in controlled conditions after the liposomal formation and the extrusion. This finding indicated that the size and charge of the liposomes remained unaffected, and it offered the opportunity for future scale-up of the whole process in view of the massive dosage needed for in vitro or in vivo applications.

#### 3.1.2. Solvent Exchange Technique for Iron-Doped Zinc Oxide Nanoparticles Coating

**Figure 6:**
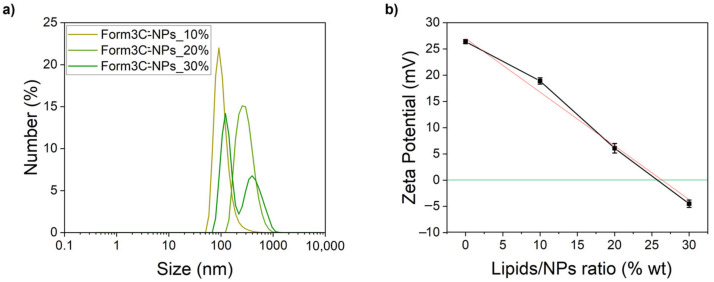
DLS size measurements (**a**) and trend of the zeta potential values (**b**) of the Form3C^−^-NPs depending on the employed lipids/NPs ratio.

As previously outlined, the negatively charged Form3C^−^ was chosen to coat our positively charged zinc oxide nanoparticles. The whole procedure was finely tuned over time to find the best conditions to obtain a stable shell on the surface of the NPs. As diffusely reported in various works on the subject [40,41,42], the main underlying mechanism of the lipidic shell formation on the surface of the NPs is the minimization of the Gibbs free energy of the lipids, previously dispersed as disassembled monomers or conventional micelles in the organic solvent mixture. When the aqueous buffer, in this case, bidistilled water, is suddenly added to the lipidic mixture, the lipids reorganize themselves and spontaneously self-assemble into various structures. Among these structures, lipid bilayers on the available substrates (in the present case, the hydrophilic surface offered by zinc oxide NPs) are the most energetically favorable [43]. To achieve this goal, the amount of water added to the lipidic mixture was finely tuned to be equal to at least 10 times the volume of the organic solvent. Finally, the first sonication step performed during the procedure was introduced to guarantee a good and homogeneous dispersion of the organic phase around the NPs before the addition of water. The second one was meant to reduce the size of the so-formed Form3C^−^-NPs. The lipid coating process using Form3C^−^ was repeated employing different lipid/NP weight ratios, and DLS and zeta potential measurements were carried out on the obtained nanoconstructs to check their stability in aqueous media and assess their surface charge. As shown by the results of Figure 6a, depending on the weight ratio between lipids and nanoparticles, different values of size and zeta potential were obtained. In particular, with increasing amounts of lipids, the zeta potential of the coated nanoparticles shifted towards lower values. Additionally, a higher degree of aggregation was evidenced by an increase in both the PDI and the size values (Table 3). Figure 6b shows a plot displaying the trend of the zeta potential values at these different lipid/NP ratios. The values were shown to have a linear relationship, further proven by means of a simple linear regression trendline (visible in red in Figure 6b, while the green horizontal one highlights the 0 mV value). This way, by imposing a desired markedly negative zeta potential value (around −25 mV as the optimum ideal value), useful to avoid aggregation in biological media [44,45], the most appropriate lipids/NPs weight ratio was easily determined. For the following experiments, the weight of lipids added to the nanoparticles was always half of the NPs’ weight (so a lipids/NPs ratio of 50% wt).

**Figure 7:**
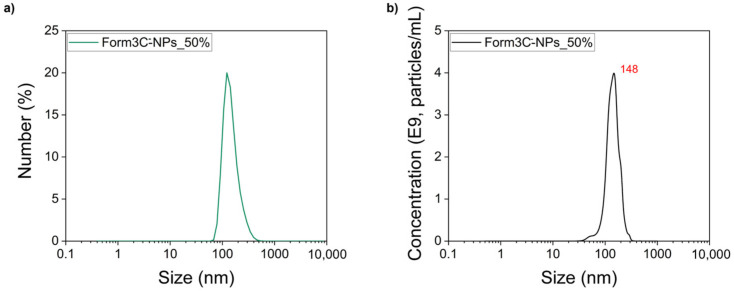
DLS (**a**) and NTA (**b**) measurements of the Form3C^−^-NPs obtained employing the optimized lipid/NPs ratio of 50% wt.

The whole coating procedure was then repeated as previously described. The DLS and zeta potentials were measured again in water. The obtained results are displayed in Figure 7a and Table 4. An extremely low value of PDI was reported, in accordance with the pronounced negative value of the zeta potential, which prevented aggregation due to the electrostatic interactions caused by the presence of the charged lipids in the shell. Moreover, the shift of the zeta potential towards negative values was the first tangible proof of the successful coating of the nanoparticles with the lipidic shell, which was further assessed via other techniques described in the subsequent sections. To confirm the DLS analysis, a NTA measurement was performed as well. The results shown in Figure 7b and reported in Table 4 were consistent with those obtained with the DLS. The NTA measure of Form3C^−^-NPs can be directly compared to that of the naked NPs, displayed in Figure S2, to appreciate the enhancement of the NPs’ dispersion in water due to the presence of the lipidic shell. 

Based on these results, the definitive coating protocol was finally established. This lipids/NPs ratio was kept unaltered throughout the rest of this study for all the following experiments. Moreover, to assess the influence of some process parameters on the solvent exchange technique, a series of experiments were carried out, which are reported in Figure S3 and Table S2.

**Figure 8:**
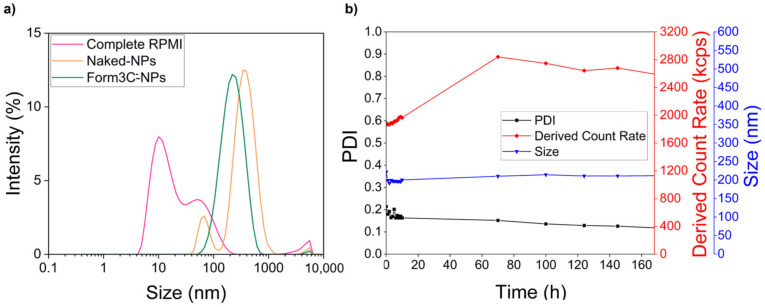
(**a**) DLS size measurements of complete RPMI, naked NPs, and Form3C^−^-NPs analyzed in RPMI; (**b**) Size, PDI, and derived count rate values of Form3C^−^-NPs measured in RPMI over time.

One of the issues related to the use of inorganic nanoparticles is their tendency to aggregate in biological media. Therefore, an experiment was carried out to prove the ability of the lipid coating to improve the NPs’ dispersion in RPMI 1640 medium supplemented with 10% heat-inactivated FBS. First, 1 mL of simple RPMI was analyzed with the DLS as a baseline. Then, 100 μg of naked NPs were dispersed in 1 mL of RPMI and analyzed. Finally, 100 μg of lipid-coated NPs were dispersed in the same volume and analyzed. As shown by Figure 8a and Table 5, the signal related to simple RPMI was very low in terms of both size and derived count rate. This reflects the presence of just the FBS content (proteins, lipids, growth factors, and anyway filtered with a 0.1 μm pore filter as stated by the manufacturer). However, when naked NPs were dispersed in RPMI, an important degree of aggregation was evidenced by both the PDI and the derived count rate values. Instead, in the presence of the lipid coating, the PDI was markedly reduced, and the aggregation rate was far lower, as witnessed by the increased value of the derived count rate. These results were stable even up to 1 week, as shown in Figure 8b, which reports the size, PDI, and derived count rate values of the Form3C^−^-NPs measured in RPMI over time.

These results proved that the presence of the lipidic coating on the NPs could markedly reduce their aggregation in biological media, increasing their biocompatibility and stability over time. Moreover, switching to a strongly negative surface charge could enhance the NP’s hemocompatibility in view of in vivo applications since blood is known to coagulate on positively charged surfaces and biomaterials [46]. It is now well established from a variety of studies that the surface charge of NPs can strongly influence their biodistribution, clearance, and induced immunological response, together with the formation of the protein corona [47,48,49,50]. In addition, electrostatic interaction between positive NPs and the well-known negatively charged cell membranes can enhance their non-specific uptake, increasing their toxicity to healthy tissues and the target ones [51]. By coating them with our Form3C^−^ lipidic shell, these risks can be significantly reduced in favor of a more controlled administration to target cells. This can be achieved via functionalizations by targeting peptides or fragments of antibodies incorporated in the lipidic shell or even pH-responsive lipids such as acid-liable PEG. These modifications promote tumor accumulation and the smart release of theranostics or immunotherapeutic agents [52].

### 3.2. Cytotoxicity Study on BxPC-3 Cells

**Figure 9:**
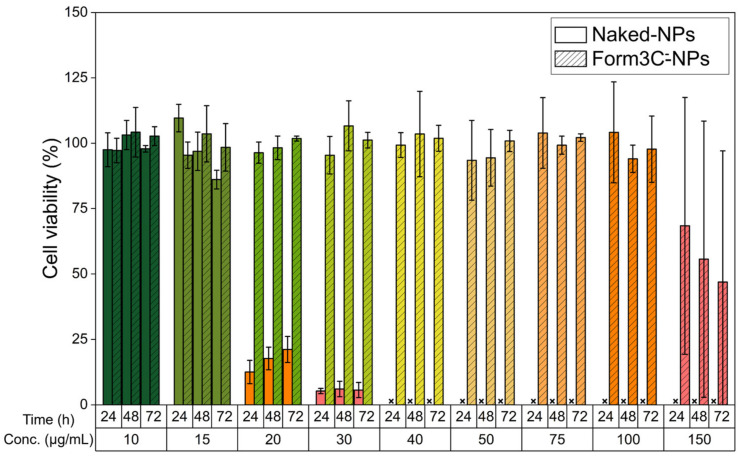
Viability of BxPC-3 cells treated with increasing amounts of both naked and Form3C^−^-coated NPs. x = data not produced due to complete cell death from 40 μg/mL of administered naked NPs.

As shown by Figure 9, when administered to BxPC-3 cells, naked NPs caused marked toxicity at 20 μg/mL at all time steps (data were not produced at 40 μg/mL since all cells were dead at this concentration). In comparison, lipid-coated NPs were proven to be safe up to 100 μg/mL and started to show signs of toxicity only at 150 μg/mL. Therefore, the presence of the lipidic shell astonishingly improved cell viability. The possible explanation for this phenomenon is the shielding effect of the lipidic coating, which prevents zinc dissolution in toxic Zn^2+^ ions and increases the biostability of the NPs. Indeed, several lines of evidence suggest that the main mechanism of zinc oxide toxicity is the release of Zn^2+^ ions, whose uncontrolled increase above the physiological threshold causes the disruption of zinc homeostasis and important damage to mitochondria and other cellular compartments [53,54]. Therefore, the main ways of regulating zinc oxide cytotoxicity consist of fine-tuning its dissolution rate via surface functionalization or coatings and the introduction of crystal defects achieved by doping [30,55]. To produce our Form3C^–^-NPs, we implemented both strategies: iron doping and functionalization with amino-propyl groups [28]. Subsequently, we coated the NPs with the lipidic shell. The observed increase in cell viability reported here is strong proof of the mitigation of zinc oxide toxicity. Cytotoxicity studies on healthy human pancreatic duct epithelial (HPDE) cells (H6c7, CVCL_0P38, from Kerafast) were also performed and are reported in Figure S4 of the Supplementary Information.

### 3.3. Colocalization with Fluorescence Microscopy

Another technique employed to assess the successful lipid coating of the nanoparticles was the qualitative colocalization study via fluorescence microscopy. This exploits the incorporation of the fluorescent functional lipid into the lipidic shell. 

**Figure 10:**
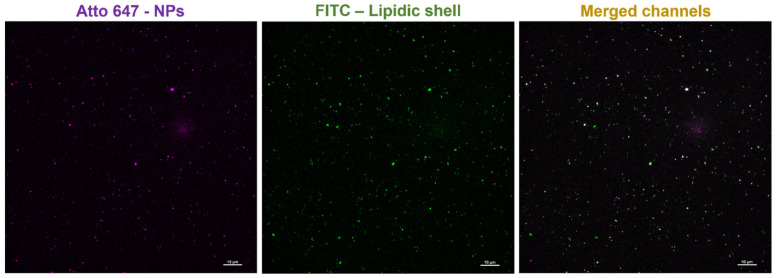
Fluorescence microscopy images showing the colocalization of the signal corresponding to the NPs (Atto647, far red channel) with the signal corresponding to the lipidic shell (FITC, green channel) in the merged channels on the right. Scale bars are set to 10 μm.

As shown in Figure 10, the signal of the far-red channel shows the NPs previously labeled with Atto647. The signal from the green channel showing the FITC dye attached to the CKAAKN peptide and conjugated to the lipidic shells was almost fully colocalized (colocalization percentage of 94%), as visible in the merged channel.

The zeta potential shift to negative values, the decreased hydrodynamic size and PDI values, the enhanced stability in biological media, and the acquired stability over time of the Form3C^−^-NPs compared to naked NPs, together with the colocalization study, all confirmed the undeniable presence of the lipidic shell on the NPs’ surface. 

### 3.4. Cytotoxicity Study with Targeting Peptide

**Figure 11:**
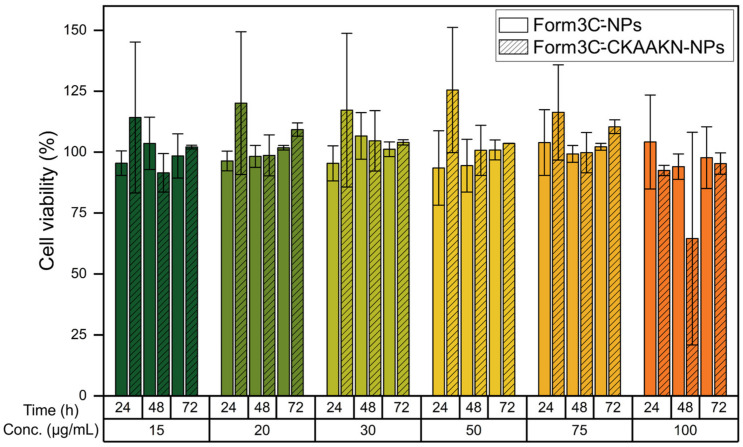
Viability of BxPC-3 cells treated with increasing doses of Form3C^−^-NPs in the presence of the targeting peptide CKAAKN, compared to the same NPs without the targeting peptide incorporated in the lipidic shell.

A second cytotoxicity study meant to compare the naked and the Form3C^−^-coated NPs in the presence of the targeting peptide was then carried out employing the same protocol previously reported. As shown in Figure 11, naked nanoparticles killed almost all cells at a concentration of 30 μg/mL. In contrast, when coated with the lipidic shell, even in the presence of the targeting peptide, cell viability was almost 100% with respect to the control up to 75 μg/mL. At 100 μg/mL, cell viability was still more than 50% for all time steps but started to show a decrease. Therefore, it can be concluded that the presence of the targeting peptide, incorporated via the functional lipid in the lipidic shell, did not affect the toxicity of the NPs towards BxPC-3 cells. 

### 3.5. Flow Cytometry and Internalization in BxPC-3 Cells

The Form3C^−^-NPs concentration selected to perform the uptake experiment was 50 μg/mL to minimize the risk of any cell death while administering a conspicuous amount of NPs to be detectable via flow cytometry. It should be noted that, compared to naked NPs, this dose was already more than three times the previous tolerated threshold. Therefore, it represents a remarkable improvement with respect to past experiments [28].

**Figure 12:**
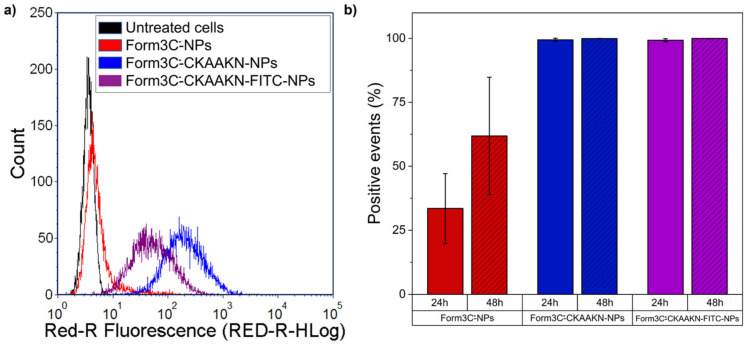
(**a**) Representative histogram of the fluorescence intensity of cells measured through the cytofluorimetric assays performed to assess Form3C^−^-NPs internalization and (**b**) BxPC-3 cells measured as positive events due to the internalization or immobilization at the outer cell membrane of Form3C^−^-NPs.

As reported in Figure 12, Form3C^−^-coated NPs already showed a slightly enhanced internalization compared to control cells (untreated). This enhancement is expressed in terms of % of positive events, indicating cells that were able to internalize or immobilize Form3C^−^-NPs at the outer cell membrane. In the presence of the targeting peptide, however, the internalization rate was astonishingly higher. It is worth mentioning that there was a slight difference in terms of fluorescence intensity retrieved by cells treated with the CKAAKN peptide and the CKAAKN-FITC one. This suggests a higher uptake for the system without the dye. A possible explanation for that might be that some of the sites of the peptides are occupied by the FITC dye attached to them, reducing their targeting ability and therefore causing a slightly lower rate of cell internalization. 

### 3.6. Fluorescence Microscopy

**Figure 13:**
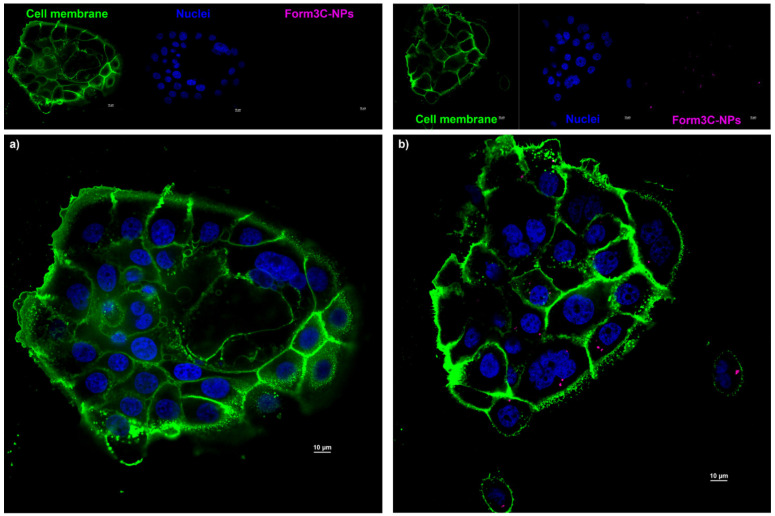
Fluorescence microscopy images of (**a**) the control cells (incubated in a complete medium without NPs) and of (**b**) cells incubated in a complete medium containing 50 μg/mL Form3C^−^-CKAAKN-NPs. Scale bars are set to 10 μm.

To further assess the presence of the NPs inside the cells and not just on the cell membrane, z-stack images through the *Z* axis of the samples were collected and the 3D reconstructions of such images are reported in Figure S5). As shown in Figure 13b, extracted from an intermediate slice of the previously mentioned z-stack collection of images, the presence of the nanoparticles inside the cell membranes can be appreciated in the picture reporting all three merged channels (blue for nuclei, green for cell membranes, far red for Form3C^−^-coated NP). As expected, the control cells (Figure 13a), not treated with Form3C^−^-CKAAKN-NPs, did not show any fluorescent signals in the far red channel. It must be noted that the selected dose of Form3C^−^-CKAAKN-NPs (50 μg/mL) was perfectly tolerated by cells, which did not show any morphological change or sign of cell death after the administration and the incubation.

### 3.7. Hemocompatibility Test

**Figure 14:**
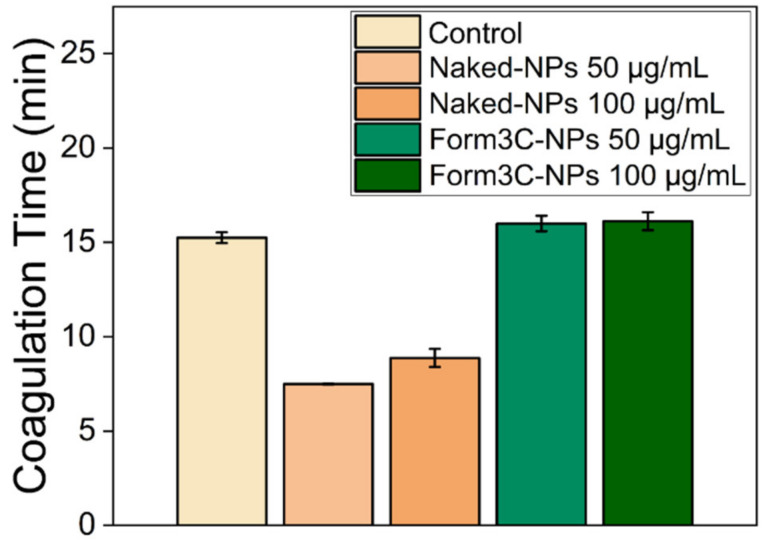
Coagulation times (min) of plasma in the presence of physiological solution (control), naked NPs, and Form3C^−^-NPs, after the addition of calcium chloride.

The results of the hemocompatibility test, which was performed to evaluate the time necessary for plasma to clot in the presence of NPs, are reported in Figure 14. Plasma citrate was used to simulate the use of anticoagulants to prevent blood clotting, and calcium chloride (CaCl_2_) was added to induce rapid calcification, manifested by an increase in turbidity in the course of approximately 15 min. Both our safe doses of Form3C^−^-NPs (50 µg/mL) and a concentration equal to twice the safe one (i.e., 100 µg/mL) were employed and compared. In this way, a general idea can be obtained for planning future in vivo tests, including higher doses of NPs than those administered to cells in vitro to achieve their therapeutical goal in much more complex systems. Compared to naked NPs (orange bars in Figure 14), the presence of the lipidic shell (green bars) had the effect of inducing coagulation at nearly the same time as the pure citrate plasma treated with physiological solution (control sample in Figure 14) after the addition of CaCl_2_. Therefore, it can be concluded that the lipidic shell essentially counteracts the tendency of blood to clot when in contact with positively charged naked NPs. These results are consistent with the points made in Section 3.1.2 about the importance of a negatively charged lipid coating on the surface of the NPs. They can constitute an optimal starting point for future in vivo validations of our nanoconstructs, suggesting that their employment in intravenous injection would not constitute a hazard even in the presence of high doses of NPs.

## 4. Conclusions

The aim of this work was to develop a customizable lipidic shell meant to be applied as a carrier for liquid moieties and drugs or as a coating for inorganic nanoparticles. Based on the same principle of electrostatic interaction as the solid lipid nanoparticles employed to carry mRNA in the newly diffused COVID vaccines, our lipidic formulations are designed to interact with their charged cargo. When employed as a smart coating for charged metal oxide nanoparticles, in the present case of iron-doped zinc oxide nanoparticles, they provide them with better dispersion in biological media and high stability over time. The administration of Form3C^−^-coated NPs to BxPC-3 cells in the proof-of-concept study resulted in a massive increase in their biocompatibility compared to pristine NPs. This enabled an escalation of the safe dose that could potentially be administered to cells in view of future theranostic treatments. Moreover, the high degree of customization of the lipidic formulation is achievable through the incorporation of a functional lipid linked to peptides or even antibody fragments. This makes it suitable for a plethora of different applications that range from tumor targeting to immunotherapy. Finally, increasing the safe NPs dose can improve the outcome of many therapies based on the stimulation of previously administered nanoconstructs via ultrasound or light irradiation. Overall, the results of the chemical characterizations and the preliminary cell culture experiments support the use of these lipidic formulations as a solid starting point for much more complex systems, thanks to their high versatility and enormous potential in future multimodal cancer therapy applications.

## Figures and Tables

**Table 1:** Lipids and molar ratios of the three realized formulations.

	Formulation 2C		Formulation 3C^+^		Formulation 3C^−^	
	Lipids	Molar Ratio	Lipids	Molar Ratio	Lipids	Molar Ratio
**Charged lipids**	/	/	DOTAP	50%	DOPA	50%
**Neutral lipids**	DOPC	60%	DOPC	10%	DOPC	10%
**Cholesterol**	Cholesterol	38.5%	Cholesterol	38.5%	Cholesterol	38.5%
**PEGylated lipids**	DSPE-PEG (2000)-Amine	1.5%	DSPE-PEG (2000)-Amine	1.5%	DSPE-PEG (2000)-Amine	1.5%

**Table 2:** DLS size, zeta potential, and NTA measurements of the three lipidic formulations before and after the extrusion.

	Formulation 2C		Formulation 3C^+^		Formulation 3C^−^	
	DLS Size		DLS Size		DLS Size	
	**Size Average**	**PDI**	**Size Average**	**PDI**	**Size Average**	**PDI**
Before extrusion	407.7 nm	0.731	216.5 nm	0.437	371.4 nm	0.527
After extrusion	138.7 nm	0.144	151.2 nm	0.106	150.7 nm	0.148
	**Zeta Potential**		**Zeta Potential**		**Zeta Potential**	
	Zeta Potential	SD	Zeta Potential	SD	Zeta Potential	SD
Before extrusion	1.6 mV	1.4 mV	57.5 mV	2.7 mV	−58.5 mV	4.3 mV
After extrusion	2.0 mV	0.4 mV	47.9 mV	1.3 mV	−51.9 mV	0.4 mV
	**NTA**		**NTA**		**NTA**	
	Mean	SD	Mean	SD	Mean	SD
After extrusion	122.4 nm	24.8 nm	152.5 nm	42.6 nm	121.0 nm	29.5 nm

**Table 3:** DLS size and zeta potential values of the Form3C^−^-NPs obtained employing different lipid/NP weight ratios in the solvent exchange process.

Form3C^−^-NPs				
	DLS Size		Zeta Potential	
Lipids/NPs Ratio	Zeta Average	PDI	Zeta Potential	SD
**10% wt**	197.2 nm	0.212	18.9 mV	0.6 mV
**20% wt**	368.0 nm	0.164	6.0 mV	0.9 mV
**30% wt**	572.5 nm	0.292	−4.5 mV	0.7 mV

**Table 4:** DLS size, zeta potential, and NTA measurements of the Form3C^−^-NPs obtained employing the optimized lipid/NPs ratio of 50% wt. * = Derived Count Rate.

	DLS Size			Zeta Potential		NTA	
Lipids/NPs Ratio	Size Avg	PDI	DCR *	Z-Pot	SD	Mean	SD
**50% wt**	203.2 nm	0.125	2624.7 kcps	−20.4 mV	0.4 mV	156.0 nm	42.8 nm

**Table 5:** Zeta Average, PDI, and derived count rate of complete RPMI, naked NPs in RPMI, and Form3C^−^-NPs in RPMI. * = Derived Count Rate.

	Zeta Average	PDI	DCR *
**Complete RPMI**	16.15 nm	0.440	49.6 kcps
**Naked NPs**	275.0 nm	0.306	1713.2 kcps
**Form3C^−^-NPs**	199.4 nm	0.181	2620.6 kcps

## Data Availability

All necessary data are included in this manuscript. Data is unavailable due to privacy restrictions.

## Supplementary Materials

The following supporting information can be downloaded at: https://www.mdpi.com/article/10.3390/nano13152250/s1, Table S1: Schematic content of Figure 5, comparing the zeta average, PDI, and zeta potential of the liposomes before and after the extrusion and after 10 days of storage; Figure S1: Process scheme of the preparation of the Form3C^−^ lipidic formulation with the incorporation of the functional lipid, in this case, either DSPE-PEG(2000)-CKAAKN or the fluorescent DSPE-PEG(2000)-CKAAKN-FITC; Figure S2: NTA measurement of naked NPs in bidistilled water; Figure S3: DLS size and zeta potential measurements of the Form3C^−^-NPs realized by removing different amounts of ethanol to assess its influence in the lipid coating process; Table S2: Schematic content of Figure S3, reporting the derived count rate of each sample; Figure S4: Viability of HPDE cells treated with increasing amounts of both naked and Form3C^−^-coated NPs; Figure S5: 3D reconstructions of BxPC-3 spinning disk confocal fluorescence microscopy images at different focuses of (a) the control cells (incubated in complete medium without NPs) and (b) cells incubated in complete medium containing 50 μg/mL Form3C^−^-NPs. Scale bars are set to 10 μm.
