# Supplementary material for: Lipidic Formulations Inspired by COVID Vaccines as Smart Coatings to Enhance Nanoparticle-Based Cancer Therapy
Source: Nanomaterials (Basel). 2023 Aug 3;13(15):2250. doi: 10.3390/nano13152250 (PMC10420688; doi:10.3390/nano13152250)
Supplement: Supplementary file 1 [file nanomaterials-13-02250-s001.zip › nanomaterials-2488578-supplementary.pdf]

Lipidic Formulations Inspired by COVID Vaccines as Smart Coatings to Enhance Nanoparticle-Based Cancer Therapy

Marzia Conte, Marco Carofiglio, Giada Rosso and Valentina Cauda *

Department of Applied Science and Technology, Politecnico di Torino, Corso Duca degli Abruzzi 24, 10129 Torino, Italy; marzia.conte@polito.it (M.C.); marco.carofiglio@polito.it (M.C.); giada.rosso@polito.it (G.R.)
* Correspondence: valentina.cauda@polito.it. Tel.: +39-011-0907389

1. Stability studies of liposomes

	Size Average			
	Before extrusion		After extrusion	
	Day 0	Day 10	Day 0	Day 10
**Form2C**	407.7 nm	760.6 nm	138.7 nm	133.1 nm
**Form3C$^+$**	216.5 nm	222.6 nm	151.2 nm	152.9 nm
**Form3C$^-$**	371.4 nm	361.3 nm	150.7 nm	143.2 nm

	PDI			
	Before extrusion		After extrusion	
	Day 0	Day 10	Day 0	Day 10
**Form2C**	0.731	0.954	0.144	0.129
**Form3C$^+$**	0.437	0.407	0.106	0.132
**Form3C$^-$**	0.527	0.531	0.148	0.121

	Zeta Potential			
	Before extrusion		After extrusion	
	Day 0	Day 10	Day 0	Day 10
**Form2C**	1.6 mV	1.3 mV	2.0 mV	0.6 mV
**Form3C$^+$**	57.5 mV	61.0 mV	47.9 mV	44.4 mV
**Form3C$^-$**	-58.5 mV	-56.0 mV	-51.9 mV	-46.7 mV

Table S1. Schematic content of Figure 5, comparing the zeta average, PDI, and zeta potential of the liposomes before and after the extrusion and after 10 days of storage

2. Optimized coating process with incorporation of functional lipid

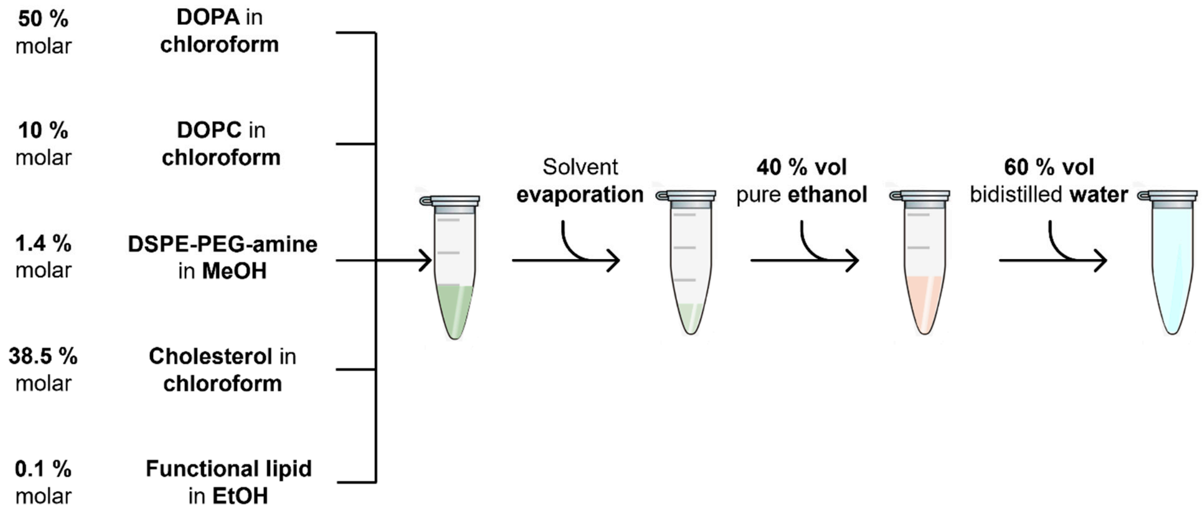

Figure S1. Process scheme of the preparation of the Form3C$^-$ lipidic formulation with the incorporation of the functional lipid, in this case, either DSPE-PEG(2000)-CKAAKN or the fluorescent DSPE-PEG(2000)-CKAAKN-FITC.

3. **NTA measurement of naked nanoparticles**

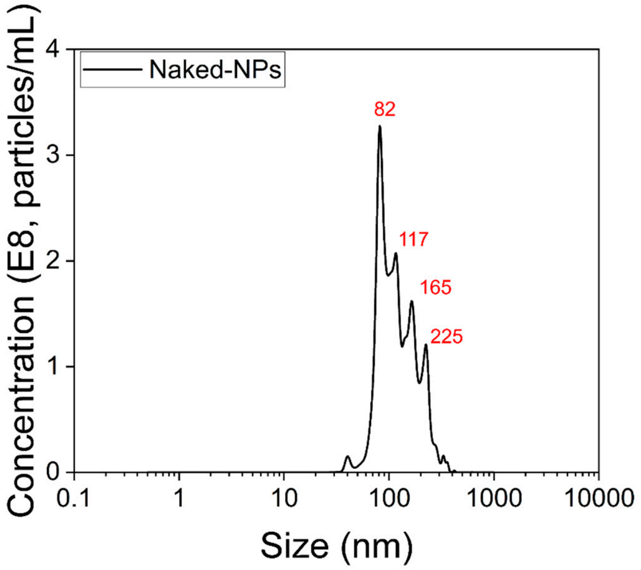

Figure S2. NTA measurement of naked NPs in bidistilled water

4. **Optimizations to improve the reproducibility of the coating process**

A series of experiments were performed to reduce, as much as possible, the experimental errors related to the coating process. Indeed, it was noticed that one of the most critical steps of the procedure was the correct removal of ethanol from the centrifuged NPs. Bearing this in mind, we simulated three different scenarios: In sample a), we removed ethanol from the NPs until they were completely dry, perturbing the pellet and removing part of it in the process; In sample b), we kept an excess of ethanol with the nanoparticles, applying a more conservative approach; in sample c), we took away as much ethanol as possible without perturbing the pellet, as typically done in the optimized protocol. Afterward, we performed the previously reported coating process on all samples and analyzed the samples in terms of hydrodynamic radius and zeta potential.

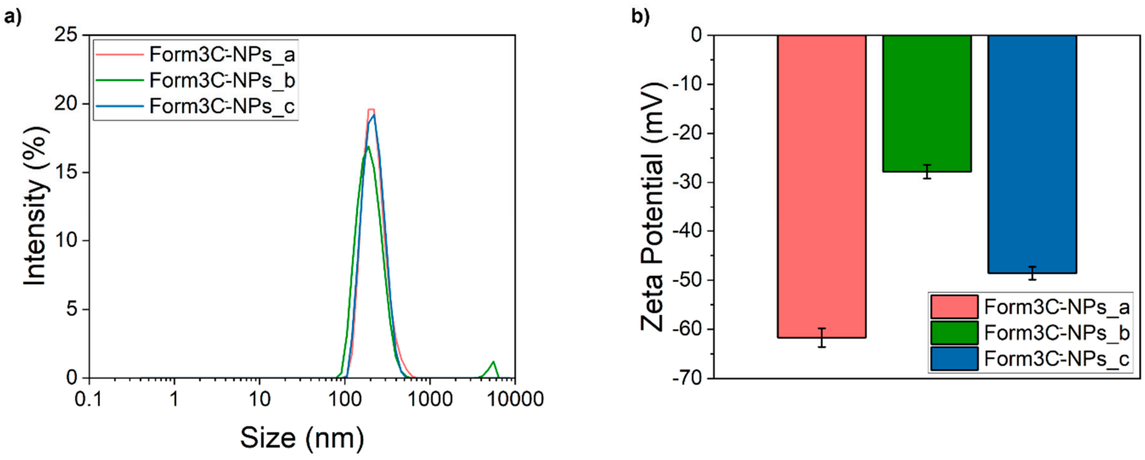

Figure S3. DLS size and zeta potential measurements of the Form3C$^-$-NPs realized by removing different amounts of ethanol to assess its influence in the lipid coating process.

As displayed in Figure S3 and reported in Table S2, removing ethanol in different ways had some tangible effects on the final nanoconstructs. Removing an excess of ethanol resulted in a loss of nanoparticles from the pellet (sample a), which caused an extremely low value of zeta potential after the coating process and a lower value of the derived count rate. Removing too little ethanol (sample b) preserved the amount of nanoparticles in the pellet. It resulted in a more modest value of zeta potential of the coated NPs, coupled with the highest value of the derived count rate. Finally, the optimized protocol resulted in an intermediate value of both zeta potential and derived count rate, implying that only a small amount of NPs is lost in the process and in the best value of PDI, resulting in a better overall dispersion of the nanoconstructs in water.

From these results, it was concluded that the best compromise to obtain highly reproducible nanoconstructs was to avoid perturbing the pellet of NPs at all costs since the whole lipid coating

process is specifically designed and tuned to a precise ratio between NPs and lipids, which ensures the best outcome in terms of efficient shell formation on top of the NPs.

	Size Average	PDI	Zeta Potential	Derived Count Rate
**Form3C$^-$-NPs_a**	244,2 nm	0,271	-61,7 ± 1,9 mV	1171,6 kcps
**Form3C$^-$-NPs_b**	197,7 nm	0,200	-27,8 ± 1,4 mV	1435,5 kcps
**Form3C$^-$-NPs_c**	218,5 nm	0,173	-48,6± 1,3 mV	1328,1 kcps

Table S2. Schematic content of Figure S3, reporting the derived count rate of each sample

5. Cytotoxicity study on healthy cells

We performed cytotoxicity assays on healthy cells, in particular healthy human pancreatic duct epithelial (HPDE) cells (H6c7, CVCL_0P38, from Kerafast), comparing the naked NPs with the lipid-coated NPS, in particular using the Form 3C$^-$.

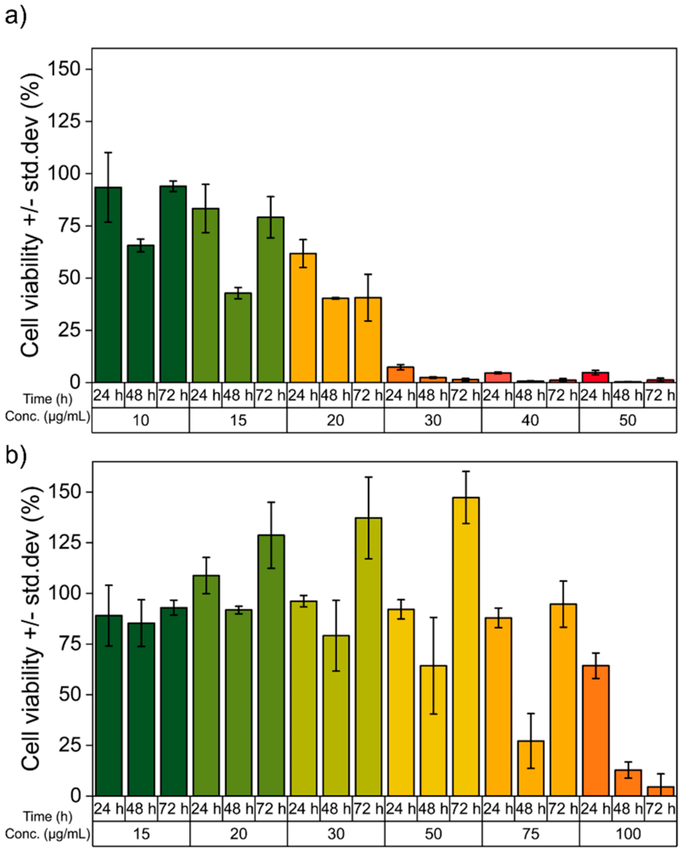

Figure S4. Viability of HPDE cells treated with increasing amounts of both naked and Form3C$^-$-coated NPs.

As shown, naked NPs are toxic starting at 30 ug/mL for all time steps (a). However, when coated by the Form3C$^-$ lipidic formulation, a remarkable increase in cell viability can be appreciated (b). Overall, this study confirms that the nanoconstructs do not kill indistinctively healthy cells. On the contrary, they are well tolerated, thanks to the presence of the lipidic shell. Moreover, since our final goal is an antitumoral effect elicited by further stimulation of the nanoparticles once localized in the tumor site, we are not bothered by the slight reduction in cell viability that healthy cells show in comparison to BxPC-3 cells. Finally, although the 48 h time point might suggest higher toxicity of the nanoconstructs, cells seem to recover after a further 24 h, as demonstrated by the high percentages of cell viability after 72 h.

6. 3D rendering of spinning disk confocal fluorescence microscopy images

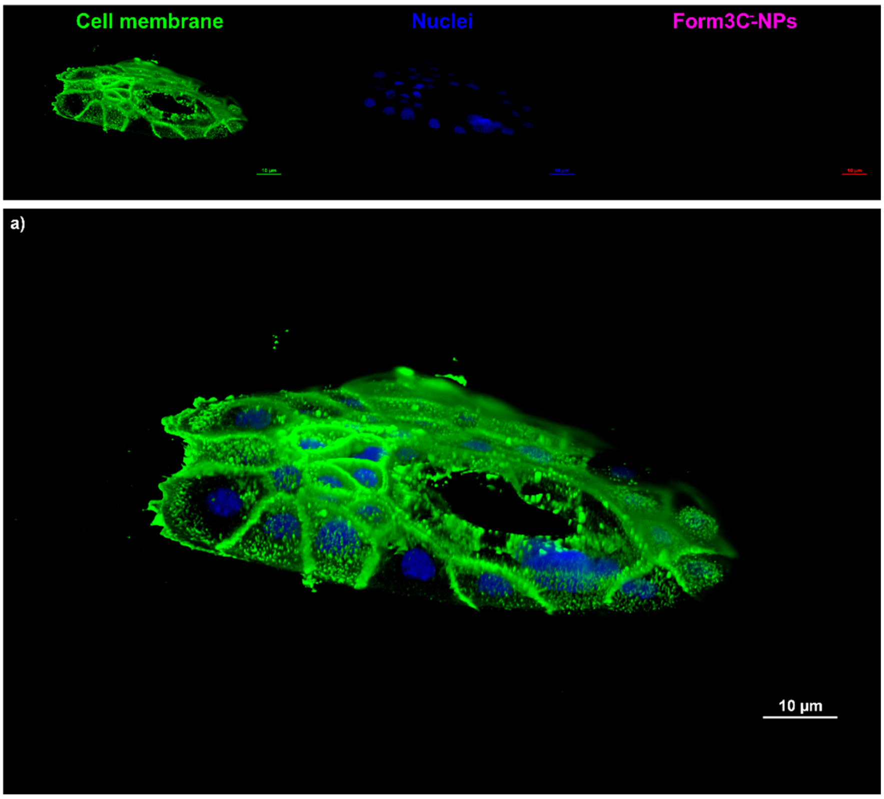

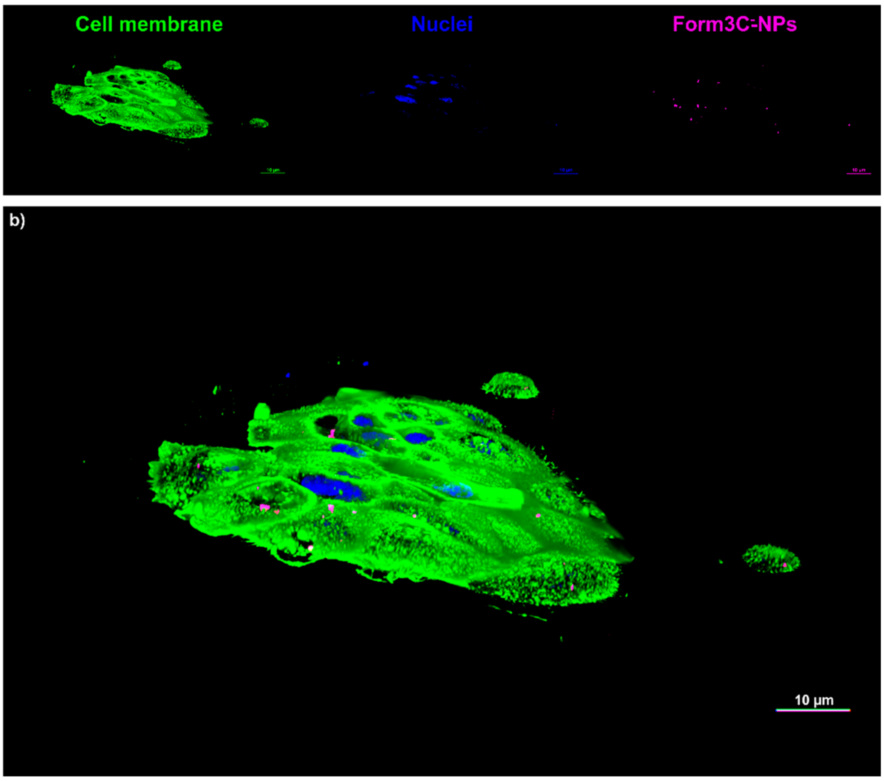

Figure S5. 3D reconstructions of BxPC-3 spinning disk confocal fluorescence microscopy images at different focuses of a) the control cells (incubated in complete medium without NPs) and b) cells incubated in complete medium containing 50 µg/mL Form3C$^-$-NPs. Scale bars are set to 10 µm.
